# Is endolymphatic sac surgery an efficient treatment of Menière's disease patients? A systematic literature search and meta-analysis

**DOI:** 10.1007/s00405-022-07580-8

**Published:** 2022-10-08

**Authors:** Franziska A. Szott, M. Westhofen, S. Hackenberg

**Affiliations:** Department of Otolaryngology-Head and Neck Surgery, RWTH Aachen University Hospital, Pauwelsstr. 30, 52074 Aachen, Germany

**Keywords:** Menière's disease, Vertigo, Endolymphatic surgery, Systematic review, Meta-analysis

## Abstract

**Purpose:**

The purpose of this work is to assess the treatment efficiency of endolymphatic sac surgery in patients with Menière's disease.

**Methods:**

This study provides a systematic literature review and meta-analysis based on the guidelines of the Cochrane Collaboration and the Preferred Reporting Items for Systematic reviews and Meta-Analyses protocol. The main inclusion criteria of the literature review were the classification categories according to the American Academy of Otolaryngology-Head and Neck Surgery guidelines of 1985 and 1995.

**Results:**

An effect of 9.25 dB postoperative weighted average hearing loss in surgically treated individuals is classified as "clinically not significant" according to American Academy of Otolaryngology-Head and Neck Surgery 85/95. In contrast, the deterioration by 26.23% in speech comprehension is considered as "clinically significant." An improvement of functional level scale assessment by two categories and vertigo control by assessment in category B can be observed.

**Conclusion:**

The findings of this meta-analysis indicate that endolymphatic sac surgery may be beneficial as a treatment for Menière's disease in terms of a positive impact on functional level scale and vertigo control while yielding a negative impact on pure tone average hearing loss and on speech comprehension.

## Introduction

Menière's disease is one of the major vestibular dysfunctions, which was first described in 1861 by the French physician Prosper Menière [1]. As an idiopathic condition, it is characterized by three typical symptoms: recurrent spontaneous episodes of rotational vertigo, hearing loss, and tinnitus with or without aural fullness.

Despite extensive data in the literature, Menière's disease remains a complex disorder of the inner ear and controversial due to its etiology, pathophysiology, diagnostics, and efficiency of treatment procedures [2].

In the surgical treatment of Menière's disease, procedures can be divided into destructive and non-destructive therapies, due to their effect on the vestibular function [3, 4]. One possible treatment option is the non-destructive endolymphatic sac surgery (ELS). ELS prevalence varies as treatment option of Menière's disease in practical use and is currently not recommended by Academy of Otolaryngology-Head and Neck Surgery (AAO-HNS) guidelines due to missing quality trial data [5].

ELS was described by Georges Portmann in 1927. By a retroauricular mastoidectomy, the saccus endolymphaticus was incised in order to allow drainage of endolymph [6]. This saccus endolymphaticus incision caused a reduction of endolymphatic pressure. The efficacy versus possible placebo effects still remain controversial [2, 5, 7]. To evaluate the treatment efficiency of ELS in Menière's disease, the present work provides a systematic literature review and meta-analysis.

## Methods

### Literature search

The systematic literature review and meta-analysis is based on the guidelines of the Cochrane Collaboration [8] and the protocol of Preferred Reporting Items for Systematic reviews and Meta-Analyses (PRISMA) [9]. The literature review has been conducted in three databases: PubMed, Embase, and Central, only by one independent peer. The PICO (Population: Menière's disease patients, Intervention: ELS, Comparison: changes in symptoms, Outcome: pre- and postoperative data) research question has been applied. Search terms among others include, for instance, Meniere disease, endolymphatic shunt surgery, endolymphatic sac surgery, endolymphatic decompression, endolymphatic sac enhancement, and endolymphatic duct blockage. The individual search strategy of literature (see appendix) involves syntax—the structure of the search strategy—and keywords adapted to the specific database [10]. The search is specified by using Medical Subject Headings, respectively, MeSH terms [Mesh], or Emtree terms. Parentheses and Boolean operators, like OR and AND, are applied to create conjunctions. Truncations (*) are used for word variations [11].

### Selection criteria

The present study aims to identify relevant studies for statistical analysis. The electronic database search was performed for studies published between 1985 and 2019.

Essential inclusion criteria comprise a description of the surgical procedure and a detailed, sufficient presentation of the collected patient data for statistical calculation. An unsuitable data format of the studies is converted to the required format if possible (e.g., patient data in % are converted to number of patients). In the case of missing data, no contact is made with the authors of the studies. Rather, when possible, our own calculation of data is performed to determine neglected values, e.g., the mean, standard deviations, and correlations. For statistical presentation, the raw data, such as number of patients and the test results, are statistically processed. Using the classification categories according to the AAO-HNS guidelines of 1985 and 1995 [12, 13], the surgical treatment efficiency of patients diagnosed with Menière's disease was determined by focusing on the pre- to postoperative comparison of the following endpoints: pure tone average (PTA), speech discrimination, vertigo control, and functional level scale (FLS). The long-term follow-up times of a patient’s cohort are considered. For the endpoint vertigo, follow-up in accordance with the AAO-HNS criteria as comparison up to 24 months after therapy was included. The follow-up times used for the other endpoints ranged from 12 months to 15 years. The median follow-up time for the endpoint PTA as well as FLS is 4 years and for speech discrimination 11.2 years.

Duplicated articles, multiple publications, letters, comments, literature reviews, articles without a full-text access, and full texts in a language other than German and English were likewise not considered.

Single and dual case displays as well as corpse and animal experiments were also not included.

Furthermore, publications about patients with surgical pre-treatment of the ear and articles about revision operations or about intraoperative combinations of surgical procedures were excluded as well.

Only variants of the ELS or modifications of the ELS relevant for this paper are taken into account. In this respect, surgical procedures, like "shunting" or "enhancement" of the saccus endolymphaticus, decompression of the unopened, non-incised and intact saccus endolymphaticus, or the blocking of the ductus endolymphaticus are included.

The incisions of the saccus endolymphaticus, like in study descriptions of “enhancement” or of “decompression,” are referred as “shunting” procedures in this paper.

The “decompression” procedure without opening the saccus endolymphaticus is equated as “decompression of the intact saccus endolymphaticus” in the present work. A damage of the saccus endolymphaticus, like the removal of the extraosseous part of the saccus endolymphaticus, led to exclusion in this paper.

Shunting into the subarachnoid space, epidural shunting, "inner endolymphatic valve", "endolymphatic sac ballooning", combination of the endolymphatic saccus endolymphaticus procedures, or intraoperative cortisone application were not analyzed.

### Risk of bias assessment

The study consideration of the ELS as a treatment of Menière's disease is evaluated by a risk of bias assessment using Methodological Index for Non-Randomized Studies (MINORS) [14]. This instrument is used for non- randomized and non-comparative studies. Eight items are scored from zero to two, with zero reflecting “not reported,” one “reported but inadequate,” and two meaning “reported and adequate.”

### Statistical analysis

The meta-analysis was performed with the program Revman (Review Manager 5.3) by “generic inverse method” and random-effect model. The statistical approach is chosen to ensure that no major distortions in the precision of the pooled effect estimates by comparing large and smaller study datasets are obtained. With the method the weighted mean is determined for the continuous endpoints: PTA in dB, speech discrimination in %, FLS in the ordinal scale 1–6, and vertigo control in categories (categories A–F are converted into an ordinal scale 1–6). Vertigo classification of AAO-HNS 1985 considers no classification in category F; thus, this paper automatically assigns zero patients to this category. Wherever feasible, improper data have been converted to the required format, e.g., patient data in % converted into the number of patients. The present paper equates data documenting patients as “deaf” with a 120 dB hearing result. Mean difference, standard error, and weight were calculated by the pre- to postoperative mean and standard deviation. The only exception is the endpoint vertigo. Its effect measure is calculated as the mean value since it already represents data from pre- and postoperative vertigo calculation. I^2^ = 0–40% is considered as low heterogeneity among the studies, I^2^ = 30–60% as moderate and I^2^ = 50–90% as high.

## Results

### Systematic literature review and bias assessment

**Fig. 1:**
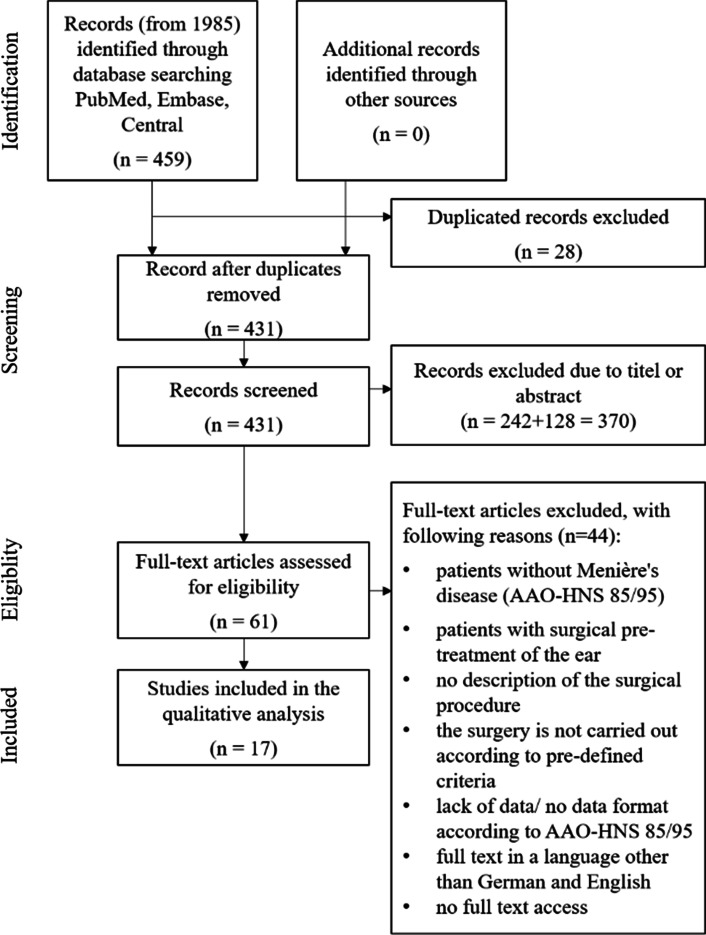
Study identification flow diagram according to PRISMA [8]

A total of 459 references were found by systematic literature search. After duplication exclusion, 431 literatures remained for title, abstracts, and full-text screening, the latter using defined selection criteria as depicted in Fig. 1. In total, 17 studies [15–31] were included.

One of these studies [16] involves both "shunting" and "decompression" procedures. Accordingly, it needs to be twice included. Studies on blocking the ductus endolymphaticus could not be detected.

Table 1 shows a risk of bias assessment using MINORS. MINORS includes the following eight items: 1. A clearly stated aim, 2. Inclusion of consecutive patients, 3. Prospective collection of data, 4. Endpoints appropriate to the aim of the study, 5. Unbiased assessment of the study endpoint, 6. Follow-up period appropriate to the aim of the study,7. Loss to follow-up less than 5%, and 8. Prospective calculation of the study size. The included studies are scored from ten to twelve, while the ideal global score is 16. None of the studies were blinded, nor prospectively calculated the study size. In contrast, all studies clearly stated their aim, used a sufficient long-term follow-up, low loss of follow-up rates, and unambiguous outcome evaluations.

**Table 1:** Methodological index for non-randomized studies

Procedure	Author	Year	1	2	3	4	5	6	7	8	Sum
Shunting	Albu	2015	2	2	1	2	0	2	2	0	11
Shunting	Brackmann	1987	2	2	1	2	0	2	2	0	11
Shunting	Brinson (*)	2007	2	2	1	2	0	2	2	0	11
Decompression	Brinson (**)	2007	2	2	1	2	0	2	2	0	11
Decompression	Gianoli	1998	2	2	2	2	0	2	2	0	12
Shunting	Jennings	1989	2	2	1	2	0	2	2	0	11
Shunting	Kim	2012	2	2	2	2	0	2	2	0	12
Shunting	Kitahara	1988	2	2	1	2	0	2	2	0	11
Decompression	Ostrowski	2003	2	2	1	2	0	2	2	0	11
Shunting	Quaranta	1998	2	2	1	2	0	2	2	0	11
Shunting	Quaranta	1997	2	1	1	2	0	2	2	0	10
Decompression	Sennaroglu	2001	2	2	2	2	0	2	2	0	12
Shunting	Thomsen	1998	2	2	2	2	0	2	2	0	12
Shunting	Tyagi	2006	2	2	1	2	0	2	2	0	11
Shunting	Welling	1996	2	2	2	2	0	2	2	0	12
Shunting	Wetmore	2008	2	2	1	2	0	2	2	0	11
Shunting	Yu	2009	2	2	1	2	0	2	2	0	11
Shunting	Zhang	2016	2	2	1	2	0	2	2	0	11

Studies marked with * in Table are considered twice

### Meta-analysis

#### Pure tone average

**Fig. 2:**
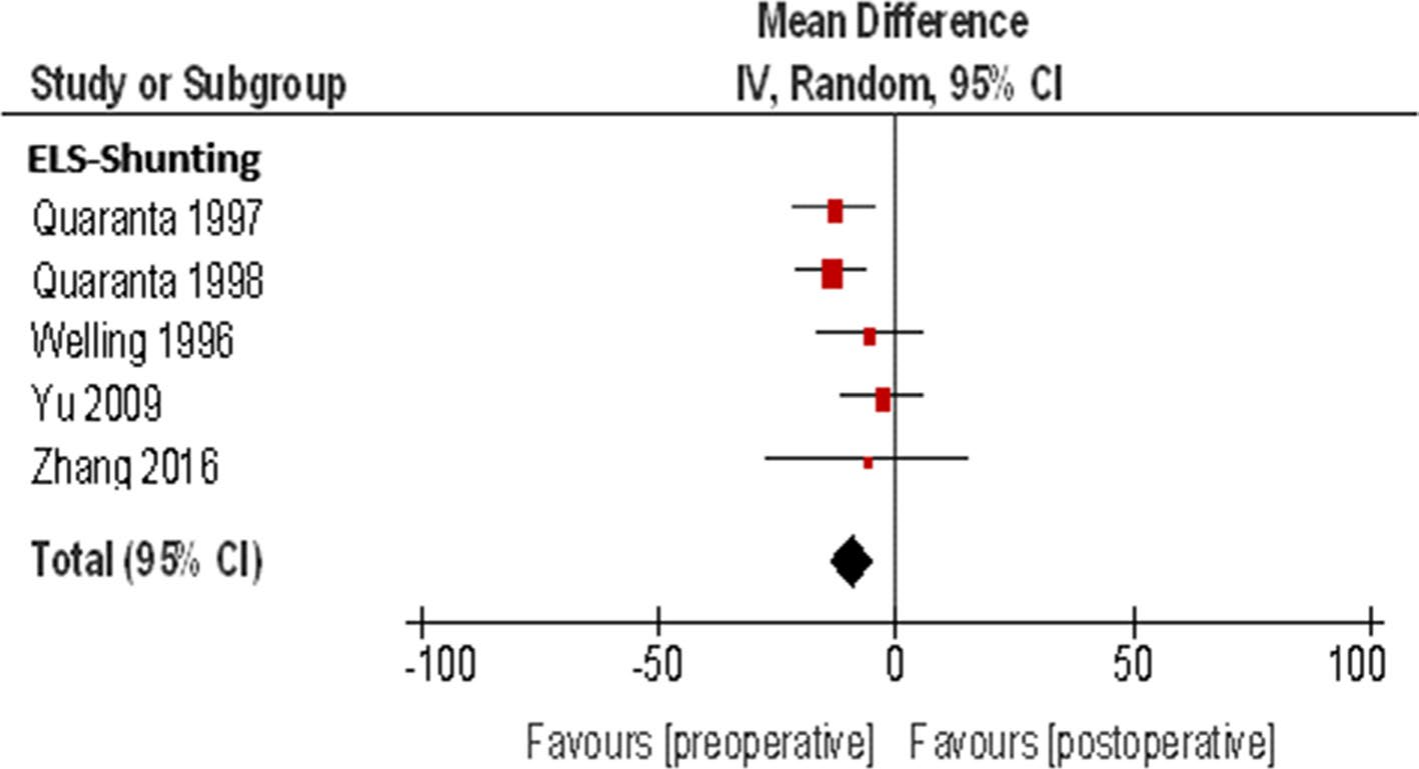
Forest plot of the ELS shunting procedure for the endpoint PTA

The meta-analysis of the ELS shunting procedure for the endpoint PTA was obtained on the base of five studies on 66 patients (Table 2) (Fig. 2). The weighted mean is − 9.25 dB (95% CI: − 13.92, − 4.57 dB, I^2^ = 12%) with a p-value of 0.0001. The p-value is less than 0.05, and therefore, the test results are considered "statistically significant." Due to AAOH-HNS 85/95, the postoperative weighted average hearing loss of 9.25 dB is "clinically not significant".

**Table 2:** Meta-analysis of the ELS shunting procedure for the endpoint PTA

		Preoperative		Postoperative					
Study or subgroup	Total	Mean	SD	Mean	SD	MD	SE	Weight (%)	Mean Difference, IV, Random, 95% CI
Quaranta 1997	17	49.41	13.46	62.24	19.21	− 12.82	4.51	23.7	− 12.82 [− 21.67, -3.98]
Quaranta 1998	18	49.44	12.42	63.11	18.28	− 13.67	3.75	32.1	− 13.67 [− 21.02, -6.31]
Welling 1996	9	45.67	20.68	51.22	30.45	− 5.56	5.73	15.6	− 5.56 [− 16.79, 5.68]
Yu 2009	16	43.81	11.40	46.63	21.44	− 2.81	4.49	23.9	− 2.81 [− 11.62, 5.99]
Zhang 2016	6	45.16	9.19	51.17	33.69	− 6.0	10.85	4.7	− 6.0 [− 27.27, 15,27]
Total (95% CI)	66							100.0	− 9.52 [− 13.92, -4.57]

Heterogeneity: Tau^2^ = 3.63; Chi^2^ = 4.57, df = 4 (*P* = 0.33); *I*^2^ = 12%

Test for overall effect *Z* = 3.88 (*P* = 0.0001)

#### Functional level scale

**Fig. 3:**
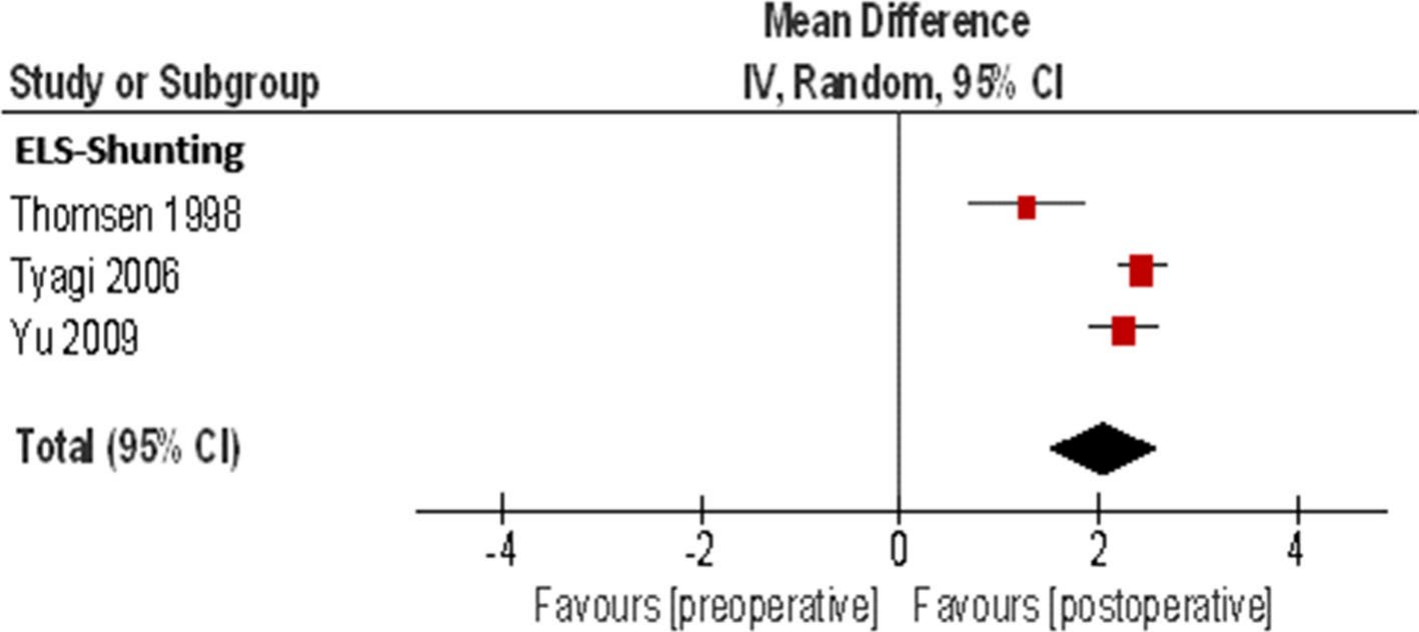
Forest plot of the ELS shunting procedure for the endpoint FLS

Three studies with 70 patients are included in the meta-analysis of the ELS shunting procedure for the endpoint FLS (Table 3) (Fig. 3). The weighted average depicts a pre- to postoperative FLS improvement of two categories, which is a weighted mean of 2.05 FLS categories (95% CI: 1.49, 2.60, *I*^2^ = 84%). The *p*-value of < 0.00001 shows "statistically significant" test results.

**Table 3:** Meta-analysis of the ELS shunting procedure for the endpoint FLS

		Preoperative		Postoperative					
Study or subgroup	Total	Mean	SD	Mean	SD	MD	SE	Weight (%)	Mean Difference, IV, Random, 95% CI
Thomsen 1998	15	4.27	1.24	3.0	1.86	1.27	0.31	27.7	1.27 [0.67, 1.86]
Tyagi 2006	39	3.67	1.07	1.23	0.62	2.44	0.13	37.6	2.44 [2.18, 2.69]
Yu 2009	16	4.81	0.73	2.56	1.27	2.25	0.19	34.7	2.25 [1.88, 2.62]
Total (95% CI)	70							100.0	2.05 [1.49, 2.60]

Heterogeneity: Tau^2^ = 0.20; Chi^2^ = 12.45, df = 2 (*P* = 0.002); *I*^2^ = 84%

Test for overall effect Z = 7.24 (*P* < 0.00001)

#### Speech discrimination

**Fig. 4:**
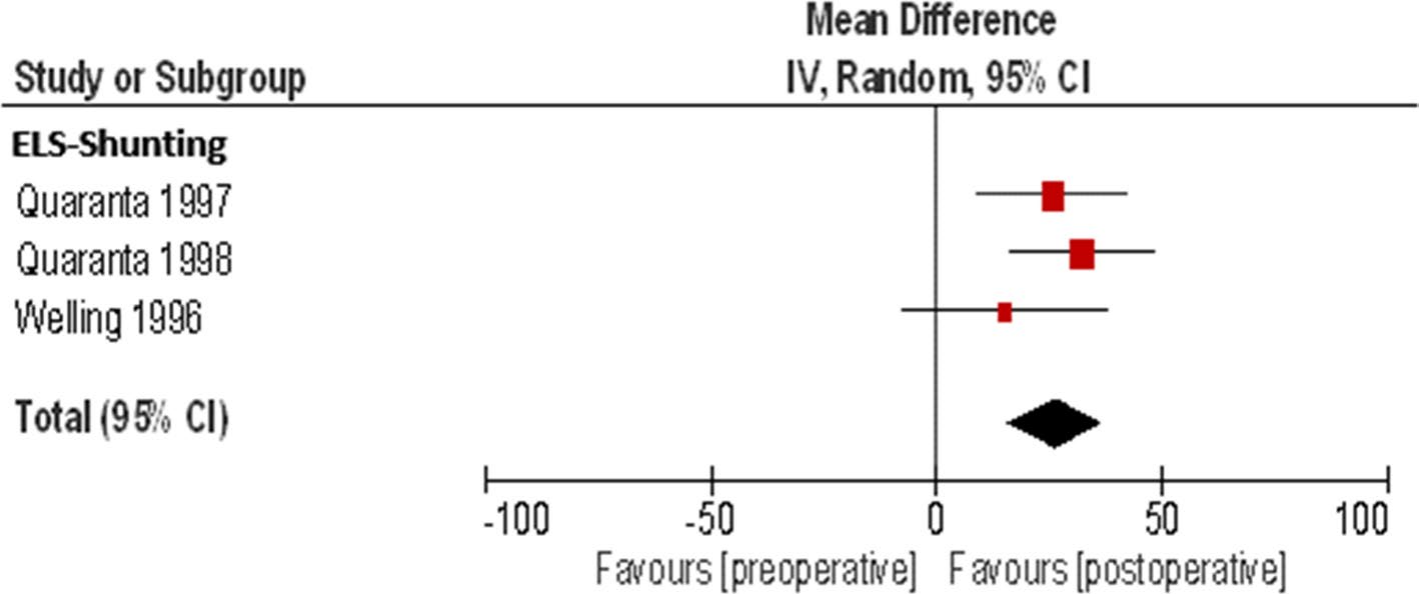
Forest plot of the ELS shunting procedure for the endpoint speech discrimination

Speech discrimination was reported in three studies with a total of 44 patients. The meta- analysis (Table 4) (Fig. 4) results in a "statistically significant" test result (p < 0.00001), without evidence of heterogeneity and shows with a weighted mean of 26.23% (95% CI: 15.89, 36.57, I^2^ = 0%) a "clinically significant" deterioration in speech comprehension according to AAO-HNS 85/ 95.

**Table 4:** Meta-analysis of the ELS shunting procedure for the endpoint speech discrimination

		Preoperative		Postoperative					
Study or subgroup	Total	Mean	SD	Mean	SD	MD	SE	Weight (%)	Mean Difference, IV, Random, 95% CI
Quaranta 1997	17	80.94	18.46	55.29	30.12	25.6	8.51	38.4	25.60 [8.91, 42.29]
Quaranta 1998	18	85.56	10.66	53.33	29.63	32.2	8.19	41.5	32.20 [16.16, 48.24]
Wlling 1996	9	80.67	21.38	65.56	34.21	15.1	11.76	20.1	15.10 [-7.96, 38.16]
Total (95% CI)	44							100.0	26.23 [15.89, 36.57]

Heterogeneity: Tau^2^ = 0.00; Chi^2^ = 1.43, df = 2 (*P* = 0.49); *I*^2^ = 0%

Test for overall effect *Z* = 4.97 (*P* < 0.00001)

#### Vertigo control

**Fig. 5:**
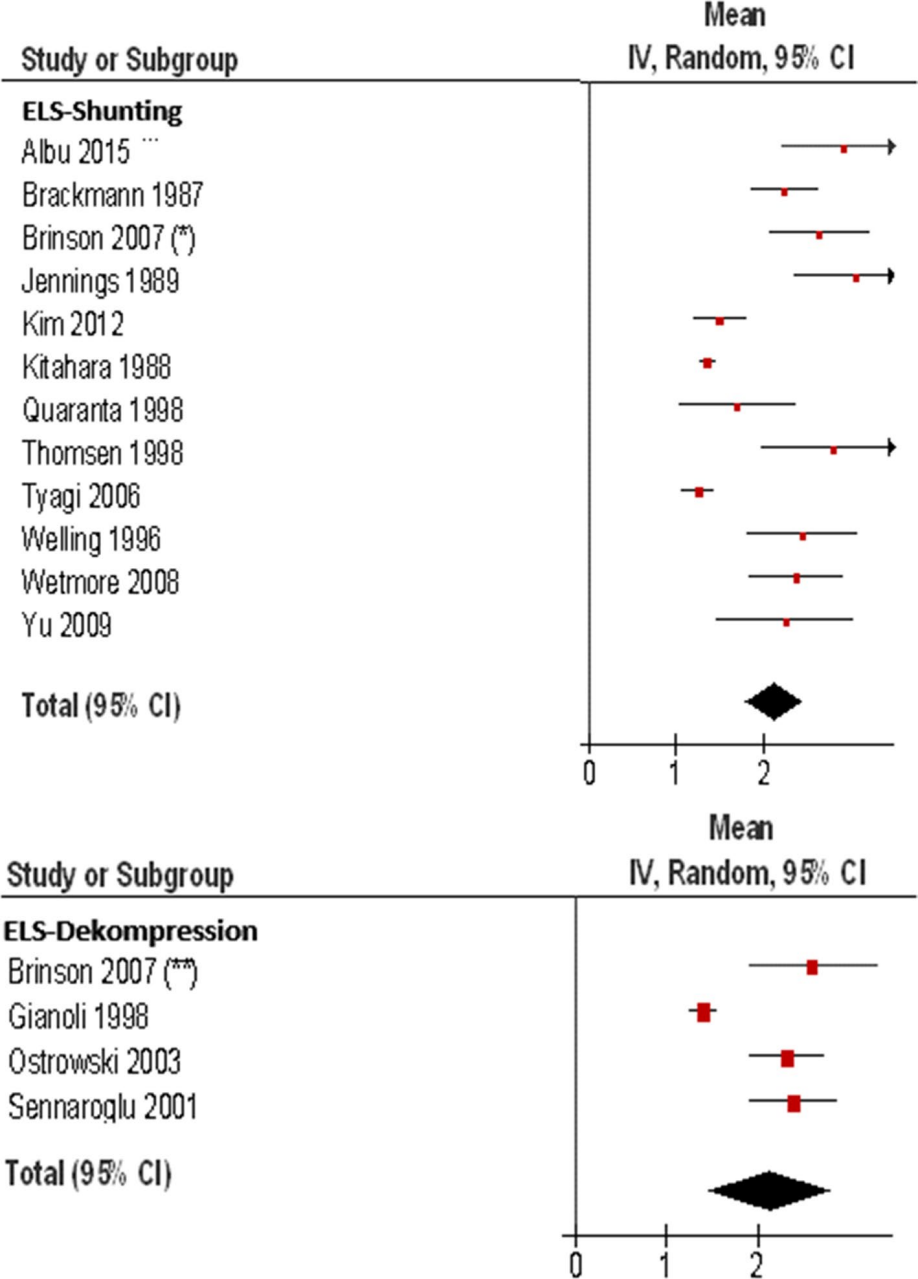
Forest plot of the ELS shunting and decompression procedure for the endpoint vertigo control. Forest plot of the ELS- decompression procedure for the endpoint vertigo control. 1: studies marked with * are considered twice

Twelve studies with 580 patients for ELS shunting and four studies with 156 patients for ELS decompression met the criteria and were included into the meta-analysis for the endpoint vertigo control (Table 5A, B) (Fig. 5). ELS shunting has a weighted mean of 2.13 in the number scale (95% CI: 1.80, 2.45, *I*^2^ = 90%) with *p* < 0.00001 and ELS decompression has a weighted mean of 2.15 in the number scale (95% CI: 1.47, 2.82, *I*^2^ = 91%) with a *p* < 0.00001. The weighted averages of both procedures show approximately the value “2” in the numerical scale, which accounts to category B if transferred to vertigo categories. Thus, a noticeable vertigo improvement can be recognized.

**Table 5:** **A** Meta-analysis of the ELS shunting procedure for the endpoint vertigo control. **B** Meta-analysis of the ELS decompression procedure for the endpoint vertigo control

Study or subgroup	Total	A	B	C	D	E	F	Mean	SE	Weight (%)	Mean, IV, Random, 95% CI
Albu 2015	34	15	5	2	1	2	9	2.91	0.37	7.1	2.91 [2.19, 3.63]
Brackmann 1987	43	19	8	4	11	1	0	2.23	0.20	9.5	2.23 [1.84, 2.62]
Brinson 2007(*)	44	20	7	6	1	0	10	2.64	0.30	8.1	2.64 [2.05, 3.22]
Jennings 1989	17	5	1	2	6	3	0	3.06	0.37	7.1	3.06 [2.34, 3.78]
Kim 2012	16	9	6	1	0	0	0	1.5	0.15	10.0	1.50 [1.20, 1.80]
Kitahara 1988	276	204	56	6	9	1	0	1.36	0.04	10.9	1.36 [1.27, 1.44]
Quaranta 1998	20	15	2	1	0	0	2	1.7	0.34	7.5	1.70 [1.03, 2.37]
Thomsen 1998	15	5	3	0	5	1	1	2.8	0.42	6.4	2.80 [1.97, 3.63]
Tyagi 2006	39	32	4	3	0	0	0	1.26	0.09	10.6	1.26 [1.07, 1.44]
Welling 1996	9	1	5	1	2	0	0	2.44	0.32	7.8	2.44 [1.82, 3.07]
Wetmore 2008	51	27	12	0	1	0	11	2.37	0.28	8.4	2.37 [1.83, 2.92]
Yu 2009	16	7	4	3	0	0	2	2.25	0.40	6.7	2.25 [1.47, 3.03]
Total (95% CI)	580									100.0	2.13 [1.80, 2.45]

B											
Brinson 2007 (**)	28	12	6	3	0	2	5	2.61	0.36	21.7	2.61 [1.90, 3.32]
Gianoli 1998	35	21	14	0	0	0	0	1.4	0.08	27.9	1.40 [1.24, 1.56]
Ostrowski 2003	68	32	17	6	2	0	11	2.32	0.22	25.6	2.32 [1.90, 2.75]
Sennaroglu 2001	25	8	5	8	2	2	0	2.4	0.25	24.8	2.40 [1.92, 2.88]
Total (95% CI)	156									100.0	2.15[1.47, 2.82]

Studies marked with * in Table are considered twice

Heterogeneity: Tau^2^ = 0.25; Chi^2^ = 110.02, df = 11 (*P* < 0.00001); *I*^2^ = 90%

Test for overall effect *Z* = 12.91 (*P* < 0.00001)

Heterogeneity: Tau^2^ = 0.42; Chi^2^ = 35.09, df = 3 (*P* < 0.00001); *I*^2^ = 91%

Test for overall effect *Z* = 6.20 (*P* < 0.00001)

## Discussion

The aim of this work is to evaluate the efficiency of ELS in the treatment of patients with Menière's disease. Based on the results, it can be generally concluded that the therapy leads to evident functional level and vertigo improvement, with limitations to long-term follow-up for vertigo by considering a period of maximum two years, while a reduction in auditory abilities occurs.

In the course of further research, one previously published study by Sood 2014 [32] was identified as comparator for the present work. The meta-analysis of Sood 2014 analyses vertigo control and hearing for ELS in the short- and long-term follow-up. Its meta-analysis is performed on the basis of a variety of literature since the inclusion criteria allowed literature older than the year 1985 and includes articles based on AAO-HNS guidelines of 1972 if data were amenable for extrapolation. Sood 2014 presents its data very detailed since ELS shunting is specified in procedure with or without silastic. However, it focuses only on vertigo control and PTA and no bias assessment has been introduced.

In contrast, the present work includes articles only on recent literature from 1985 to 2019 or data based on AAO-HNS 85/95. Only the latest follow-up was considered since we were only interested if the procedure is effective in the long term. In this paper all procedures with the incision of the saccus endolymphaticus with or without silastic are referred as “shunting” procedures and no further distinction is being made. In comparison to Sood 2014, a major difference in the present work is the statistical analysis of the meta-analysis itself. Sood 2014 distinguishes vertigo control in Categories A/B or not and PTA as either “improved/stable” or “worsened”. However, in this paper, it is detailed extracted in which vertigo category the patients are defined at and it is clearly stated how much hearing loss has occurred. This paper additionally focuses at the following endpoints: speech discrimination and FLS. Thus, the following study draws more differentiated conclusions.

The lack of randomized controlled trials leads to major limitations of this work and its conclusions. To this fact, an analysis of preoperative vs. postoperative assessment results in a lower evidence base compared to a comparison of therapy vs. control group. Thus, placebo effects, the impact of natural, and unimpaired course of disease cannot be completely excluded.

In terms of data collection for meta-analysis, limited data availability and methodological challenges are faced.

Classification categories according to the AAO-HNS guidelines of 1985 and 1995 were used as criteria for endpoint evaluation. The endpoint vertigo control was included in numerous references. However, in comparison to the remaining endpoints, PTA, speech discrimination, and FLS were sparsely considered in studies. This implies a limited relevance for clinical evidence due to data inconsistency.

Further methodological difficulties are obvious upon examining the extent of heterogeneity of the studies. With corresponding heterogeneity among the studies by *I*^2^ = 12% in the meta-analysis of PTA and *I*^2^ = 0% of speech discrimination, those two endpoints show low heterogeneity. This result is emphasized by the relatively homogeneous mean average values that were recorded preoperatively. In contrast, a high degree of study heterogeneity can be observed among the studies for the FLS-endpoints. The high degree of study heterogeneity neither can be excluded for the endpoint vertigo control. This disparity may be due to different disease stages in which the patients were recorded and later treated. The ambiguity of the AAO-HNS diagnosis and classification is another reason for this apparent clinical heterogeneity. Therefore, it is uncertain whether only patients with an actual disease pattern of Menière's disease were included into the studies.

In conclusion, statements with relevance for clinical evaluations appear to have methodological limitations and thus show few evidences. To this point, there is no diagnostic test that clearly confirms Menière's disease, e.g., by biological marker or image morphological analysis. Despite extensive data in the literature, Menière's disease is still controversially discussed and treated. Thus, further fundamental research to improve the current standard of diagnosis and classification of Menière's disease is desirable. This research could be supported by high-quality trials in the future. However, the present study also shows that research in the area has been rather sparse in the last decade. Only three studies were included in the systematic review which are up to date. This could indicate that the ELS method is unlikely to prevail in practice, whereas further research could lead to an evidence-based statement on the standard of ELS use.

## Appendix

### A.1 Search strategies of the systematic literature search for Pubmed

"Meniere Disease"[Mesh] OR menieres disease [tw] AND (endolymphatic shunt surgery OR endolymphatic shunt* OR endolymphatic sac shunt* OR endolymphatic sac surgery OR endolymphatic sac decompression OR endolymphatic sac enhancement OR endolymphatic duct blockage)

### A.2 Search strategies of the systematic literature search for Embase

“meniere disease”/exp AND (“endolymphatic shunt”/exp OR “endolymphatic sac surgery”/exp OR “endolymphatic decompression” OR “endolymphatic duct blockage”)

### A.3 Search strategies of the systematic literature search for CENTRAL

#1: MeSH descriptor: [Meniere Disease] explode all trees

#2: ("Meniere disease"):ti,ab,kw

#3: #1 OR #2

#4: (endolymphatic shunt surgery OR endolymphatic sac surgery OR endolymphatic sac decompression OR endolymphatic sac enhancement OR endolymphatic duct blockage):ti,ab,kw

#5: #3 AND #4
