# Correction to: Is endolymphatic sac surgery an efficient treatment of Menière's disease patients? A systematic literature search and meta-analysis

**DOI:** 10.1007/s00405-022-07717-9

**Published:** 2022-11-09

**Authors:** Franziska A. Szott, M. Westhofen, S. Hackenberg

**Affiliations:** Department of Otolaryngology-Head and Neck Surgery, RWTH Aachen University Hospital, Pauwelsstr. 30, 52074 Aachen, Germany

## Correction to: European Archives of Oto-Rhino-Laryngology 10.1007/s00405-022-07580-8

In the original publication of the article, Heterogeneity values of Table 5A, 5B was published incorrectly. The correct values are provided below,

Heterogeneity values of Table 5A is,

**Table:** 

Heterogeneity: Tau^2^ = 0.25; Chi^2^ = 110.02, df = 11 (*P* < 0.00001); *I*^2^ = 90%
Test for overall effect *Z* = 12.91 (*P* < 0.00001)

Heterogeneity values of Table 5B is,

**Table:** 

Heterogeneity: Tau^2^ = 0.42; Chi^2^ = 35.09, df = 3 (*P* < 0.00001); *I*^2^ = 91%
Test for overall effect *Z* = 6.20 (*P* < 0.00001)

The original article has been updated.

